# A human induced pluripotent stem cell toolbox for studying sex chromosome effects

**DOI:** 10.1016/j.stemcr.2025.102678

**Published:** 2025-10-16

**Authors:** Ruta Meleckyte, Wazeer Varsally, Jasmin Zohren, Jerry Eriksson, Tania Incitti, Linda Starnes, Amy Pointon, Ryan Hicks, Benjamin E. Powell, James M.A. Turner

**Affiliations:** 1 Sex Chromosome Biology Laboratory, The Francis Crick Institute, 1 Midland Road, London, UK; 2 Max Planck Institute for Plant Breeding Research, Cologne, Germany; 3 Clinical Pharmacology and Safety Science, R&D, AstraZeneca, Gothenburg, Sweden; 4 Cell Culture & Fermentation Science, R&D, AstraZeneca, Gaithersburg, Maryland, USA; 5 Bioscience Metabolism, Early CVRM, BioPharmaceuticals R&D, AstraZeneca, Gothenburg, Sweden; 6 Clinical Pharmacology and Safety Science, R&D, AstraZeneca, Cambridge, UK; 7 BioPharmaceuticals R&D Cell Therapy Department, Research and Early Development, Cardiovascular, Renal, and Metabolism (CVRM), BioPharmaceuticals R&D, AstraZeneca, Gothenburg, Sweden; 8 School of Cardiovascular and Metabolic Medicine & Sciences, King’s College London, London, UK; 9 School of Life and Medical Science, University College London, London, UK

**Keywords:** hiPSCs, human, iPSCs, iPSC model, induced pluripotent stem cells, sex differences, sexual dimorphism, disease modeling

## Abstract

Sex chromosomes shape male (XY)-female (XX) differences in development and disease. These differences can be modeled *in vitro* by comparing XY and XX human induced pluripotent stem cells (hiPSCs). However, in this system, inter-individual autosomal variation and unstable X-dosage compensation can confound identification of sex chromosomal effects. Here, we utilize sex chromosome loss in XXY fibroblasts to generate XX and XY hiPSCs that are autosomally isogenic and exhibit stable X-dosage compensation. We also create X-monosomic (XO) hiPSCs, to investigate X-Y dosage effects. Using these autosomally isogenic lines, we examine sex differences in pluripotent stem cell expression. Transcriptional differences between XX and XY hiPSCs are surprisingly modest. However, X-haploinsufficiency induces transcriptional deregulation predominantly affecting autosomes. This effect is mediated by Y-genes with broad housekeeping functions that have X-homologs escaping X inactivation. Our isogenic hiPSC lines provide a resource for exploring sex chromosome effects on development and disease *in vitro*.

## Highlights

• Isogenic iPSC toolbox with XO, XX, XY, and XXY karyotypes to study sex chromosome effects
• Transcriptional landscape is similar in human XX and XY iPSCs
• Multiple Y chromosome genes are expressed in iPSCs
• Loss of a sex chromosome strongly impacts iPSC gene expression

## Teaser

In this article, Turner and colleagues introduce an established isogenic human iPSC resource encompassing XO, XX, XY, and XXY karyotypes to investigate sex chromosome effects. Transcriptomic profiling revealed minor differences between XX and XY iPSCs, confirmed the expression of multiple Y-linked genes, and demonstrated that loss of sex chromosome exerts a stronger impact on gene expression than chromosome gain.

## Introduction

Sexual dimorphisms are abundant in mammals. Historically, these were attributed to the distinct sex hormone milieu of males (XY) and females (XX). However, recent literature shows that sex chromosome complement can directly impact phenotypes in both healthy and disease state (Blencowe et al., 2022; Arnold et al., 2024; Aguet et al., 2020; Naqvi et al., 2019; Snell and Turner, 2018; Tallaksen et al., 2023). For instance, two X chromosomes predispose women to autoimmune disease (Brown et al., 2022), while Y chromosome loss in men is associated with cardiovascular disease, neurodegeneration, and cancer (Gutiérrez-Hurtado et al., 2024). Identifying how sex chromosome genes directly impact development and disease susceptibility in the absence of sex hormones represents a major challenge in biology and personal medicine.

Sex differences can be assayed *in vitro* by comparing XY and XX human induced pluripotent stem cells (hiPSCs) and their differentiated derivatives. However, two challenges are often encountered. First, since existing XY and XX hiPSC lines are derived from different individuals, they also carry differences in autosomal DNA sequences, which confound the dissection of sex chromosome-derived effects. Second, XX hiPSCs often exhibit instability in X-dosage compensation, leading to gene expression artifacts (Mekhoubad et al., 2012; Tomoda et al., 2012; Bansal et al., 2021; Pasque and Plath, 2015; Bar et al., 2019; Raposo et al., 2025; Brenes et al., 2021; Topa et al., 2024). In mammals, X-dosage compensation is achieved by the collaboration of two processes. X-upregulation balances expression between the X and the autosomes, while X inactivation, mediated by the non-coding RNA *XIST*, balances X-expression between the sexes (Disteche, 2016). Together, these processes result in an X-to-autosome (X:A) ratio close to 1.0. Previous work has shown that XX hiPSCs are prone to X chromosome erosion, in which *XIST* expression is lost from the inactive X, and previously silent X-genes are consequently reactivated (Mekhoubad et al., 2012; Tomoda et al., 2012; Bansal et al., 2021; Pasque and Plath, 2015; Bar et al., 2019; Raposo et al., 2025; Brenes et al., 2021; Topa et al., 2024). An ideal hiPSC resource for dissecting sex differences must therefore comprise XX and XY hiPSCs that are both autosomally isogenic and exhibit stable X-dosage compensation.

Autosomally isogenic hiPSCs have been generated previously, providing important insights into the impact of sex chromosome complement on pluripotent stem cell transcription. One study reported the generation of autosomally isogenic XY and XXY hiPSCs from non-mosaic XXY (Klinefelter syndrome) fibroblasts, but XX hiPSCs were not derived (Fiacco et al., 2021). Another study generated XXY, XY, and XX autosomally isogenic hiPSCs, but the status of X-dosage compensation was not assayed in detail (Waldhorn et al., 2022). Further reports have compared either XX or XY cells to autosomally isogenic XO (Turner syndrome) cells; but in these instances, the parental XX cells were not isogenic to the XY cells (Ahern et al., 2021; Urbach and Benvenisty, 2009).

In this study, we report the generation of XX and XY hiPSCs that are autosomally isogenic and exhibit stable X-dosage compensation. Using our cell lines, we show that XX and XY hiPSCs exhibit surprisingly similar gene expression profiles. By generating XO hiPSCs, we also show that the Y chromosome and inactive X chromosome exert considerable effects on pluripotent stem cell gene expression.

## Results

### Generation and characterization of autosomally isogenic XXY, XX, XY, and XO hiPSCs

**Figure 1:**
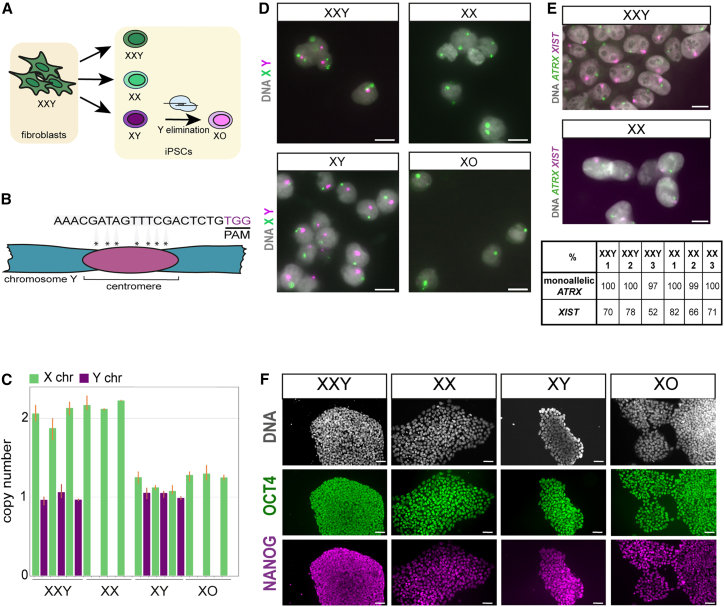
Autosomally isogenic hiPSC generation and characterization (A) Schematic view of hiPSC generation through XXY fibroblast reprogramming and Y-elimination via CRISPR-Cas9. (B) Schematic view of seven sgRNA guide target sites on the Y centromere. Protospacer adjacent motif (PAM) is indicated. (C) qPCR copy number variation analysis using TaqMan *AR* (X chromosome) and *SRY* (Y chromosome) gene probes in hiPSC clones, with standard error shown in orange. (D) Sex chromosome DNA-FISH of isogenic XXY clones (*n* = 129), XX (*n* = 74), XY (*n* = 119), and XO (*n* = 88) hiPSCs by X and Y DNA-FISH. The Y chromosome was present in 90%, 97%, 88%, 92%, 94%, and 89% of XXY 1 (*n* = 129), XXY 2 (*n* = 103), XXY 3 (*n* = 67), XY 1 (*n* = 119), XY 2 (*n* = 250), and XY 3 (*n* = 119) lines, respectively. (E) Representative *XIST* and *ATRX* RNA FISH analysis shows *XIST-*positive cells present in 78% and 82% of XXY 2 and XX 1, respectively. Table below shows percentage of *XIST*-positive cells in XXY 1 (*n* = 64), XXY 2 (*n* = 71), XXY 3 (*n* = 60), XX 1 (*n* = 57), XX 2 (*n* = 298), and XX 3 (*n* = 84) hiPSCs. (F) OCT4 and NANOG protein expression in hiPSCs. The brightness has been increased in XXY and XO images to improve visualization. Scale bars in (D) and (E), represent 10 μm; in (F), 50 μm.

To generate hiPSC clones with distinct sex chromosome complements but identical autosomes, we subjected non-mosaic XXY fibroblasts to reprogramming, which promotes sex chromosome loss (Hirota et al., 2017). We used integration-free reprogramming to avoid potential transgene expression artifacts (Beltran et al., 2020). Y chromosome loss gave rise to XX hiPSCs, while X chromosome loss generated XY hiPSCs (Figure 1A). Subsequently, we used CRISPR-Cas9-mediated Y chromosome elimination to generate XO from XY hiPSCs (Figure 1A; Adikusuma et al., 2017). We achieved this using a guide RNA that targets a repeat region on the Y chromosome centromere (Figures 1B and S1A). In the resulting XO cells, we tested the top five potential off-target sites and did not observe off-target mutations (Figure S1B).

Copy number variation qPCR and DNA fluorescence *in situ* hybridization (FISH) were used to establish the sex chromosome complement of our XXY, XX, XY, and XO hiPSCs (Figures 1C and 1D). In addition, karyostat analysis was employed to confirm that they were autosomally euploid (Figure S1C). SNP analysis verified that all XY clones derived from XXY fibroblasts carried the same parental X chromosome (Figure S1D). We first assayed X inactivation in our XXY and XX hiPSC clones by assessing allelic balance for X-genes with informative SNPs. This analysis revealed that X inactivation was efficient for these genes, with all reads derived from one X chromosome (Figure S1D). We also screened all XXY and XX hiPSC clones for X chromosome erosion, using a previously described approach combining RNA-FISH for *XIST* and the X-inactivated gene *ATRX* (Tchieu et al., 2010), only retaining lines that exhibited *XIST* clouds and monoallelic *ATRX* expression (Figure 1E). Additionally, we confirmed that the Y chromosome was retained in our XXY and XY hiPSC lines (see Figure 1D). We ultimately generated 12 autosomally isogenic hiPSC lines, comprising triplicates for each genotype. All hiPSC lines exhibited the expression of undifferentiated state markers POU5F1 and NANOG, as assessed by immunofluorescence (Figure 1F), as well as competence to differentiate to endoderm, mesoderm, and ectoderm lineages (Figure S1E). We used G-banding to confirm that our XXY hiPSCs carried no translocations (Figure S1F).

### Minimal gene expression differences between XX and XY iPSCs

**Figure 2:**
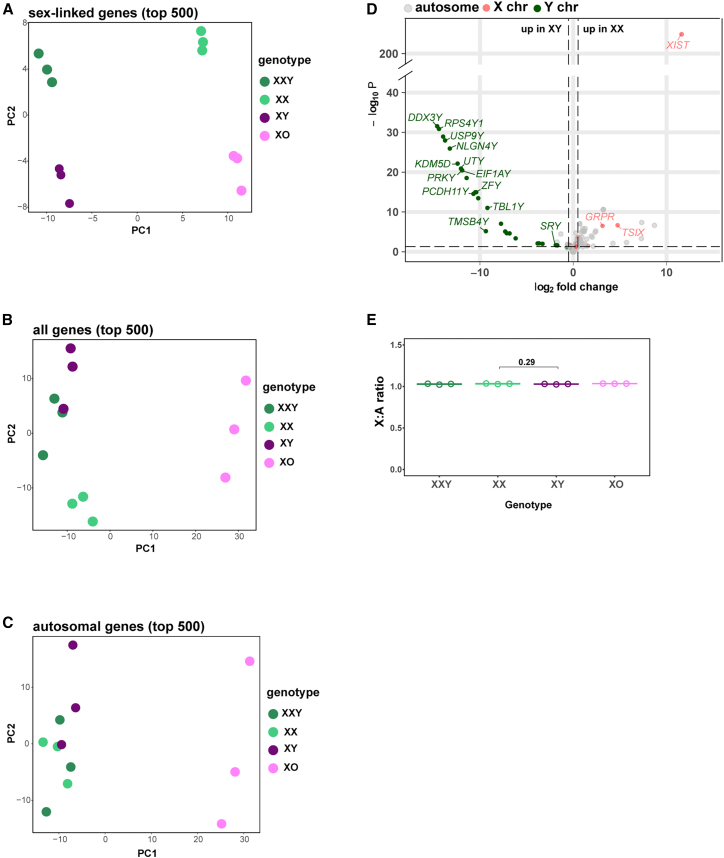
Overview of sex chromosome effects on the hiPSC transcriptional landscape (A) PCA of hiPSCs using the top 500 most variable sex-linked genes. (B) PCA of hiPSCs using the top 500 most variable genes. (C) PCA of hiPSCs using the top 500 most variable autosomal genes. (D) Volcano plot of gene expression between XX and XY hiPSCs (note split *y* axis). Horizontal dashed line represents a transformed adjusted *p* value of 0.05. Vertical dashed lines represent log_2_FC = 0.5. (E) X:A ratio in hiPSCs, *p* value was calculated using an unpaired t test.

To assay sex chromosome effects, we performed bulk RNA sequencing (RNA-seq) on our hiPSC lines. As expected, principal-component analysis (PCA) focusing on the top 500 most variable sex-linked genes showed that the lines segregated according to their sex chromosome complement (XXY, XX, XY, and XO; Figure 2A). We then repeated the PCA using the top 500 most variable genes derived from all genes. We found that XXY, XX, and XY hiPSCs were more closely clustered to each other than to the XO hiPSCs (Figure 2B). This observation was retained when we used only the most variable autosomal genes (Figure 2C). These findings demonstrate that sex chromosomes impact autosomal genes in *trans* and that X chromosome monosomy has a strong impact on autosomal gene expression (as discussed later).

We next examined differentially expressed (DE) genes between XX and XY hiPSCs. We observed a modest effect, with only 82 genes differentially expressed (log_2_FC0.5, *p*adj < 0.05; Figure 2D), indicating that the transcriptomes of XX and XY hiPSCs are similar (Table S1A). This finding demonstrates that the Y chromosome and inactive X chromosome have a similar impact on the transcriptome of XY and XX hiPSCs, respectively. Among the 82 DE genes, 33 were sex linked (26 Y-linked, 7 X-linked; Table S1A). Of the 26 Y-linked genes, 13 were protein-encoding, and the remainder were novel transcripts or pseudogenes. The protein-coding Y genes comprised 48% of the total protein-coding gene families on the Y chromosome (13 out of 27 genes) (Rhie et al., 2023). XX hiPSCs exhibited higher expression of the known X-inactivation escapees *XIST*, *GRPR*, *KDM6A*, and *SYAP* (Wainer Katsir and Linial, 2019; Topa et al., 2024; Raposo et al., 2025), as well as *TSIX*, *CA5B*, and *PAICSP7.* In contrast, most upregulated genes in XY hiPSCs (87%) were Y-linked (Figure 2D). These genes, including the testis-determining factor *SRY*, were enriched for regulatory functions, e.g., chromatin modification, RNA stability, and translation, and have been shown to exhibit strong purifying selection during evolution (Bellott et al., 2014; Cortez et al., 2014).

The finding that our XX and XY hiPSCs exhibited few DE genes indicated that, in both genotypes, X-dosage compensation was complete. We confirmed this using analysis of X:A ratios. In both the XX and XY hiPSCs, the X:A ratio was close to 1.0, demonstrating that they were indeed fully X-dosage compensated (Figure 2E). This finding also supported our earlier *ATRX* RNA-FISH analysis, which showed that X inactivation is robust in our XX hiPSCs (Figure 1E). Given the modest differences in gene expression, Gene Ontology analysis revealed only a few pathways differentially impacted by sex chromosome complement, with most reflecting the known functions of Y-genes, e.g., in chromatin remodeling (Figure S1G; Table S1B).

A previous study observed 313 DE genes between autosomally isogenic XX and XY hiPSCs, when employing a *p*adj < 0.1 (Waldhorn et al., 2022). When we applied the same criteria to our dataset, we observed only 162 DE genes. Only 15 genes overlapped between these two datasets, 14 of which were sex linked (Figure S2A). To understand the differences in autosomal gene expression, we compared the two datasets by PCA and Pearson correlation. PCA revealed that our XX and XY replicates (Figure 2A) showed lower gene expression variation than the Waldhorn replicates (Figure S2B). Furthermore, our dataset exhibited higher Pearson’s correlation coefficients between replicates and genotypes than the Waldhorn dataset (Figure S2C). Higher correlation coefficients between our replicates were retained when both datasets were processed together (Figure S2D).

### Sex chromosome monosomy significantly impacts hiPSC gene expression

**Figure 3:**
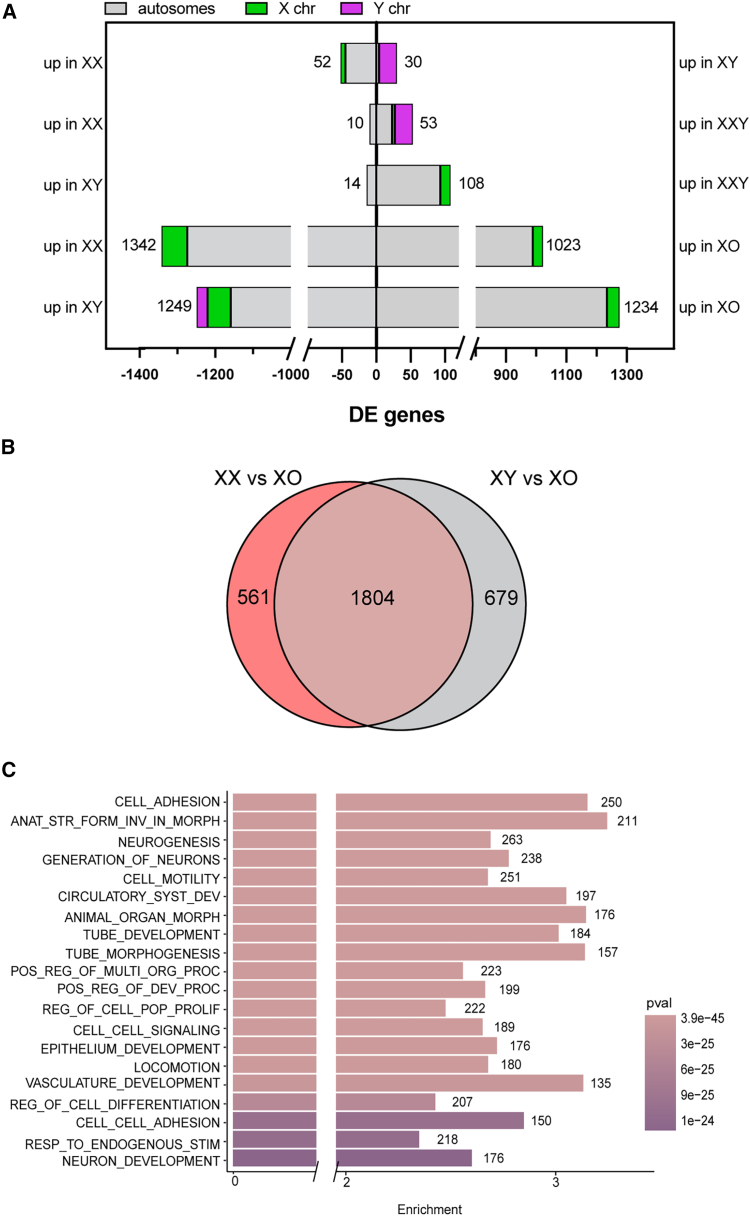
Effect of sex chromosome aneuploidy on the hiPSC transcriptional landscape (A) Bar plot of DE (log2FC ≥ 0.5, *p*adj ≤ 0.05) genes between all generated isogenic hiPSCs lines. (B) Overlap of DE genes between XX versus XO (pink circle) and XY versus XO (gray circle) comparisons. Significance of overlap was calculated as padj = 0. (C) Functional enrichment analysis of overlapping DE genes from (B).

We addressed the extent to which sex chromosome aneuploidy impacts gene expression. Both XXY and XO hiPSCs exhibited X:A ratios that were comparable to XX and XY hiPSCs (Figure 2E). Our PCA revealed that the gene expression profile of XXY hiPSCs was similar to XX and XY hiPSCs, while the gene expression profile of XO hiPSCs was more distinct (Figure 2B). This conclusion was supported by DE analysis. We identified 63 DE genes between XXY and XX hiPSCs, 41% of which were Y-linked, and 122 DE genes between XXY and XY hiPSCs, including known X-inactivation escapees (Figure 3A; Tables S1C and S1D) (Bellott et al., 2014; Berletch et al., 2011; Wainer Katsir and Linial, 2019). In comparison, a significantly larger number of genes were differentially expressed in the XO versus XY or XX hiPSC comparisons (Figure 3A; Table S1E). We found 2,483 DE genes between the XY and XO hiPSCs, indicating a strong influence of the Y chromosome on the hiPSC transcriptome. In addition, 2,365 genes were differentially expressed between the XX and XO hiPSCs (Table S1F), indicating a marked impact of the inactive X chromosome on the hiPSC transcriptome. Importantly, in both cases, the majority (95%) of DE genes were autosomal (Figure 3A). A previous study identified DE genes between XX, XY, XXY, and XO human fibroblasts (San Roman et al., 2024). Between hiPSCs and fibroblasts, there was little overlap between DE genes in like-for-like genotype comparisons (Figure S2E; Table S1G). We conclude that X chromosome monosomy causes significant transcriptional deregulation and that sex chromosomes act in *trans* to modify autosomal gene expression in a cell type-specific manner.

### The Y chromosome and inactive X chromosome have shared targets in hiPSCs

Our findings revealed that the absence of the Y chromosome or the inactive X chromosome significantly impacted gene expression in hiPSCs. Previous work found that the Y chromosome and inactive X chromosome similarly influence transcription in human somatic cells and that this effect is mediated by Y-genes with X-homologs that escape X inactivation (San Roman et al., 2023, 2024). Our earlier XX versus XY hiPSC comparison indicated that a similar phenomenon is conserved in hiPSCs. Interestingly, ten of the thirteen Y genes we found expressed in hiPSCs (Figure 2D) have X-encoded homologs that are known to escape X inactivation (*DDX3X*, *KDM5C*, *RPS4X*, *USP9X*, *NLGN4X*, *UTX*, *PRKX*, *EIF1AX*, *TBL1X*, and *ZFX*; Bellott et al., 2014; Berletch et al., 2011). To further resolve whether the Y and inactive X have overlapping functions in hiPSCs, we looked for commonality in DE genes between the XY versus XO and XX versus XO comparisons. The degree of overlap was highly significant (*p*adj = 0), comprising 73% of all XY-XO and 76% of all XX-XO DE genes (Figure 3B; Table S1H). Gene Ontology analysis identified multiple cellular pathways impacted by both Y and inactive X chromosomes, including motility, adhesion, and neurogenesis (Figure 3C; Table S1I). Additionally, we detected pathways that were uniquely altered in either the XY versus XO comparison or the XX versus XO comparison (Figures S3A and S3B; Tables S1J–S1M). These findings suggest that X-Y homologs act as dosage-sensitive regulators of the hiPSC transcriptome.

## Discussion

Sex chromosomes play a crucial role in male-female differences in health and disease, yet there is a lack of *in vitro* systems to fully investigate these effects (Kodama and Gan, 2019; Miquel et al., 2023; Wilson, 2021; Wiese et al., 2023). To address this gap, we present a set of XX and XY hiPSCs that are autosomally isogenic and exhibit stable X-dosage compensation. Using these lines, we find, in contrast to a previous study (Waldhorn et al., 2022), that male-female differences in hiPSC transcription are modest. A source of the difference is likely to be the lower replicate correlation in the Waldhorn data; this may lead to numbers of DE genes that are not truly representative of the biological differences between each genotype. Replicate variation may be linked to the fact that Waldhorn cells were derived from an XXY/XY/XX mosaic, where distinct clones may acquire genetic/epigenetic differences; variation in computational methodology such as filtering genes and DE thresholding would also impact the results. Finally, differences could arise from distinct hiPSC culture conditions in the two studies: Waldhorn et al. used feeders while we did not. The lower replicate variability in both our XX and XY hiPSC lines suggests that they represent an improved model for future studies of sex differences. By differentiating our autosomally isogenic hiPSCs to specific cell types in a 2D or 3D manner, one could investigate sex chromosome effects on health and disease phenotypes and test potential therapeutic targets associated with sex-linked genes (Qi et al., 2023; Rabeling and Goolam, 2023). It would be important in the future to generate isogenic XX and XY hiPSCs from other genetic backgrounds to complement those described here.

Dissecting the impact of sex chromosomes on sex differences can be challenging. Some effects are attributable to the presence or absence of the Y chromosome, others to X-dosage (one in males, two in females), or X-imprinting a maternal X in males, versus a maternal and a paternal X in females (Lau, 2020; Snell and Turner, 2018). Our XO hiPSCs represent a useful tool to deconvolve Y- and X-dosage effects. By comparing transcriptomes, we show that both the Y chromosome and the inactive X chromosome regulate the hiPSC transcriptome, a phenomenon that has also been described in other cell types (San Roman et al., 2024). This function is likely mediated by deeply conserved, dosage-sensitive X-Y gene pairs. Our work adds to growing evidence that the phenotypes associated with Turner syndrome result from haploinsufficiency for these ancient X-Y homologs (Trolle et al., 2016; Bellott et al., 2014; Cortez et al., 2014).

Finally, we show that X-monosomy causes significant transcriptional deregulation. This observation may be of relevance to understanding why Turner syndrome is associated with a high rate of miscarriage (Monney et al., 2000; Gravholt et al., 1996). Our XO hiPSCs will complement others (Ahern et al., 2021; Urbach and Benvenisty, 2009) as a resource with which to identify the molecular basis of Turner syndrome phenotypes in differentiated cells and tissues.

## Methods

### Human fibroblast culture

GM03102 (XXY) fibroblasts were obtained from Coriell Institute for Medical Research. Fibroblasts were cultured in fibroblast medium containing Advanced DMEM/F12 medium (Thermo Fisher Scientific, 12634010) supplemented with 20% fetal bovine serum (Biosera, cat. no. FB-1001/500).

### hiPSC generation and maintenance

Dermal fibroblasts were thawed at passage 6 and seeded at a density of 5 × 10^4^/well in 3 wells of a 6-well plate coated with 1% gelatin. Cells were plated in fibroblast medium and cultured for 48 h in 37°C, 5% CO_2_, and 21% O_2_. The CytoTune2.0 (Thermo Fisher scientific, A16517) was used to reprogram the cells following kit’s recommendation. Briefly, on the first day of reprogramming, day 0, the medium was changed and complemented with Sendai virus particles using 5:5:3 ratio. At day 7, cells were replated on vitronectin XF (STEMCELL Technologies, 7180)-coated wells, and at day 8, medium was changed to Essential 8 (Thermo Fisher Scientific, A1517001) or StemFlex medium (Thermo Fisher Scientific, A3349401). iPSC-like colonies started to appear 10–18 days following the transfection; they were manually picked and transferred to feeder-free conditions in vitronectin XF-coated 6-well plates with E8 or StemFlex medium containing 10 μM Y-27362 (Bio-Techne, 1254), respectively. The medium was changed after 24 h. Colonies were expanded by washing with DPBS (Gibco, 14190-144) and splitting at a 1:3 to 1:6 using versene (Thermo Fisher Scientific, 15040066), and single-cell cloning was used to select cells with sex chromosome loss. We have isolated five male clones and 8 female clones from 813 single-cell clones. In this study, we use XXY_1, XXY_2, XXY_3, XX_1, XX_2, XX_3, XY_1, XY_2, XY_3, XO_1, XO_2, and XO_3 cell lines. These cell lines have been registered to hPSCreg under the names accordingly: CRICKi024-A, CRICKi024-B, CRICKi024-C, CRICKi024-D, CRICKi024-E, CRICKi024-F, CRICKi024-G, CRICKi024-H, CRICKi024-I, CRICKi24-G-1, CRICKi24-H-1, and CRICKi24-I-1. Cell lines were dissociated with versene and collected in KOSR media (Thermo Fisher Scientific, 10828028) containing 10% DMSO (Merck, D2650) with cell lifters (Fisherbrand, 08-100-241). The working bank cells were thawed using 10 μM Y-27362.

### Short tandem repeat profiling

The Cell Services, a Science Technology Platform (STP) within the Francis Crick Institute, performed the short tandem repeat (STR) profiling on DNAs from the parental fibroblast sample and iPSC lines using the Powerplex 16 HS System (Promega, DC2101). Analysis was done using Applied Biosystems 3500XL genetic analyzer. All lines were sent regularly for STR profiling (every eight passages) since reprogramming started.

### Mycoplasma detection test

The Cell Services Platform (STP) at the Francis Crick Institute confirmed the absence of mycoplasma contamination using the Phoenix DX mycoplasma mix (Procomcure, PCCSKU15209) for PCR amplification. Cells were regularly sent for mycoplasma testing (every eight passages) since reprogramming started.

### Y chromosome sgRNA generation and off-target site identification

Single-guide RNA (sgRNA) AAACGATAGTTTCGACTCTGTGG was designed using publicly available CRISPOR tool (Haeussler et al., 2016). The centromere region 10350000–10450000, unique to Y chromosome, was tested, and a single sgRNA with predicted high target and low off-target activity was selected. Following a published protocol, oligonucleotides with BbsI tails were annealed and ligated into the relevant vector (Ran et al., 2013). The sgRNA oligonucleotide was sourced from Integrated DNA Technologies. We used the Cas-OFFinder tool to identify potential off-target sites (Bae et al., 2014).

### Primer design

All primer pairs used in this study were designed using the publicly available tool Primer3 (http://bioinfo.ut.ee/primer3/). All PCR amplifications were carried out using Q5 high-fidelity DNA polymerase (NEB) at recommended Q5 thermocycling conditions. To amplify off-target regions for MiSeq analysis, primers were designed using Primer3 and extended to contain MiSeq adaptor sequences (see relevant section). All primer sequences are listed in Table S2.

### Transfection assay

hiPSCs were maintained in Advanced E8 or StemFlex medium and plated as single cells a day before transfection. On day 1, cells were transfected with the Cas9-eGFP vector plasmid containing sgRNA targeting Y chromosome, Optimem (Gibco, 31985-062), and FuGeneHD buffer (Promega, E2311). Ratios were used following manufacturer’s instructions. Targeted hiPSC clones were selected by adding puromycin (0.5–1 μg/mL) when they’ve reached 80% confluency on day 2 or day 3 for 24–48 h. Targeted colonies appeared by day 6–12. Transfection efficiency is dependent on cell line and was recorded between 5% and 45%. In this study, we use XY_1, XY_2, and XY_3 cell lines, passage numbers between P10 and P15.

### Immunofluorescence assay

We used immunostaining assay to evaluate pluripotency and germ layer precursor potential. Induced pluripotent stem cells (iPSCs) (passage between P5 and P26) were fixed with 4% paraformaldehyde in DPBS (Thermo Fisher Scientific, 14190094) for 1 h at room temperature, then washed three times with PBS-A. Blocking and permeabilization were achieved by incubation with dPBS with 3% bovine serum albumin (Merck, A9647) and 1% Triton X-100 (Merck, T8787) for 1 h at room temperature. Primary antibodies were diluted in blocking solution and incubated overnight at 4°C. Following incubation, the cells underwent 2 × 5 min washes with PBS-A. Secondary antibodies were diluted 1:200 in 3% BSA and incubated for 2 h at room temperature. After 3 × 5 min washes in PBS-A, 5 μg/mL DAPI (Merck, D9542) was added for 5 min during the middle wash to perform nuclear staining. The used primary antibodies are listed in Table S3.

### Direct differentiation assay

The STEMdiff Trilineage Differentiation Kit (STEMCELL Technologies, 5230) was used to differentiate cells into three germ lineage precursors. The assay was performed as per kit instructions. We evaluated the differentiation potential of all lines by immunostaining for lineage-specific markers on day 5 (mesoderm and endoderm) and day 7 (ectoderm). Cell lines’ passage numbers were between P10 and P26 for this experiment.

### Genomic DNA isolation and copy number variation assay

Cells were collected from the well and pelleted down after centrifugation at x300g for 4 min. Cell pellet was resuspended in 200 μL of dPBS. Invitrogen PureLink Genomic DNA kit (Invitrogen, K1820-02) was used to isolate DNA following kit’s instructions. Isolated DNA concentration was measured using Nanodrop 2000c device (Thermo Scientific).

TaqMan Copy Number Assays (Thermo Fisher Scientific) were used with probes for SRY on the Y chromosome and AR on the X chromosomes (Hs01026408_cn and Hs04511283_ cn, respectively). Each sample was subjected to duplex TaqMan RT-qPCR according to Applied Biosystems protocol. All reactions were run in quadruplicates. Data were analyzed using CopyCaller version 2.1 software (Applied Biosystems). Previously established XX and XY hiPSCs were used as control samples.

### DNA-FISH

Centromeric probes for X and Y chromosome, MetaSystems XA X/Y (Pisces Scientific, D-5608-100-OG), were used to assess X and Y chromosome number in cells. Cell lines of passage between P4 and P10 were attached on glass slides by growing cells on slides (fibroblasts) or air-drying dissociated cells on slides (iPSCs). Cells were fixed in 4% paraformaldehyde in phosphate-buffered saline (PBS) at room temperature for 10 min and permeabilized in 0.05% Triton X-100 in PBS at room temperature for 10 min. Slides were washed in 2× SSC at room temperature and 75°C and denatured in 70% formamide in 2× SSC. After dehydration in an ethanol series (70, 80, 95, 100%), air-dried specimens were hybridized with the denatured probe in hybridization buffer (2× SSC, 50% formamide, 25% dextran sulfate, 5 mg/mL bovine serum albumin, 1 mM vanadyl ribonucleoside) at 37°C for overnight. After wash in 2× SSC at 45°C, 0.1× SSC at 60°C, and 4× SSC, 0.1% Tween 20 at room temperature, slides were mounted in Vectashield with DAPI (2BScientific, H-1200-10).

### RNA-FISH

Cells of passage P10–P15 were grown on slides or dissociated and air-dried on the cooled slides. Cold permabilization buffer (0.5% Triton X-100, 2 mM vanadyl ribonucleoside in PBS-A) was added on the slides for 10 min following fixation with cold 4% PFA. After removing the fixation solution, slides were rinsed in ice-cold PBS-A, dehydrated through ice-cold 70%, 80%, 95%, and 100% ethanol for 5 min each, and air dried. BAC XIST RP11-13M9 (CHORI) and BAC ATRX RP11-1145J4 (CHORI) were labeled using Nick Translation Direct Labeling Kit (Abbott Laboratories, 07J00-01) according to manufacturer’s instructions and using fluorescent nucleotides (for XIST spectrum orange – dUTP; Abbott, 02N32-050, for ATRX spectrum green – dUTP; Abbott, 02N33-050). Cells were hybridized with a denatured mix of probes along with 1 μg salmon sperm DNA in hybridization buffer (50% formamide, 25% dextran sulfate, 5 mg/mL BSA, 1 mM vanadyl ribonucleoside complex in 2× SSC) at 37°C overnight in a humid chamber. Stringency washes were performed on a hot plate, three times for 5 min in 50% formamide, 1× SSC (pH 7.2–7.4) pre-heated to 45°C, and three times for 5 min in 2× SSC (pH 7–7.2) pre-heated to 45°C. Cells were stained with DAPI in 2× SSC (1 μg/mL) for 10 min at room temperature, rinsed once with 2× SSC, and mounted in Vectashield with DAPI and stored at −20°C.

### Karyotyping and G-banding assays

The SNP-based chromosomal microarray KaryoStat assay was performed by Thermo Scientific (USA) using genomic DNA of the iPSC lines (P10–P15). XXY hiPSC G-banding analysis was performed by Cell Guidance Systems.

### Microscopy and image analysis

Immunostainings for pluripotency and trilineage markers and DNA and RNA-FISH images were acquired using the Whitefield Imaging system AxioImager.M1 (Zeiss) and MicroManager (2.0) using 10× and 40× oil objectives. 150 (FITC), 400 (Cy3), and 400 (Cy5) laser power were used for pluripotency markers; 300 (FITC), 300 (Cy3), and 300 (Cy5) used for trilineage germ layer markers; and 400 (FITC) and 400 (Cy3) were used for DNA/RNA-FISH markers. At least 50 single cells spread over 3 to 8 fields were acquired for each cell line. The number of nuclei/field was defined by DAPI-positive cells, and the percentage of positive signals for XIST and ATRX RNA-FISH, as well as X- and Y-centromeric signals for DNA-FISH, was calculated. Cells after images were processed and analyzed using ImageJ software (v.2.1).

### RNA isolation

RNA was extracted using TRI Reagent (Merck, T9424) following the chloroform-isopropanol protocol (Chomczynski and Sacchi, 2006). Briefly, the cells were pelleted and dissolved in 500 μL TRI reagent. After adding 100 μL of chloroform, cell lysate was vigorously shaken and span in cold centrifuge for 30 min. Aqueous phase was collected and precipitated using isopropanol. The pellet was dried out and dissolved in 50 μL of RNase-free water.

### Bulk RNA-Seq library preparation and sequencing

RNA libraries were generated using the KAPA mRNA HyperPrep Kit (Roche, KK8581) according to manufacturer’s instructions. Briefly, samples of passage P5–P20 were normalized to 100–1,000 ng of RNA in a final volume of 50 μL. PolyA-tailed RNA capture was performed twice with 50 μL of capture beads at 65°C for 2 min first and 70°C for 2 min for second capture following 20°C for 5 min both times. Beads were washed to remove other RNA species after each capture and eluted in 20 μL of Fragment, Prime and Elute Buffer. The fragmentation reaction was run for 6 min at 94°C for a library insert size of 200–300 bp. Fragmented RNA underwent first and second-strand cDNA synthesis according to manufacturer’s instructions. The adaptor ligation was carried out using the KAPA Unique Dual-Indexed Adapters Kit (15 μM) (Roche, KK8727) stock diluted to 1.5–7 μM. 50 μL of ligation buffer and 10 μL of DNA ligase were added to 60 μL of cDNA and incubated at 20°C for 15 min. SPRISelect beads 0.63–0.7× were used to remove short fragments. Library was amplified using 20 μL cDNA with 25 μL of Kapa HiFi HotStart PCR master mix plus 5 μL of Library Amplification Primer mix. 8–13 PCR cycles were performed as recommended by the manufacturer for a total RNA input of 100–1,000 ng. Amplified libraries were purified via a SPRISelect 1× bead cleanup. The quality and fragment size distributions of the purified libraries were assessed by a 4200 TapeStation Instrument (Agilent Technologies). Libraries were pooled and sequenced on the Illumina NovaSeq6000 in PE100 configuration to an average depth of 25M paired-end reads.

### RNA-seq data profiling

Raw RNA-seq reads were processed using the RNA-seq nf-core pipeline (v.3.14). Reads were aligned to the hg38 genome using star_rsem. Specifically, XXY and XY genotypes were aligned to the standard hg38 genome while the XX and XO genotypes was aligned to a modified hg38 genome, which did not include the Y chromosome sequence. The two resulting raw counts tables were merged. The raw counts were processed in R using the DESeq2 package (v.1.40.2). Counts were normalized using the DESeqDataSetFromMatrix() and DESeq() functions, specifying “∼genotype” in the design formula. For comparisons against the Waldhorn dataset, “∼batch + genotype” was specified as the design formula. Very lowly expressed genes were filtered out by applying a rowSums filter of ≥10 to the counts table. For the PCA plots, the DESeq2 counts were first transformed to the variance stabilizing transformation counts (VST) using the vst() function. The top 500 most variable genes from either the whole dataset, sex-linked genes, or autosomal genes were then used to generate the respective PCAs. For the PCA of the Waldhorn data, the top 500 most variable genes were obtained from only the Waldhorn dataset after processing it in DESeq2 as detailed earlier. DE genes were identified using the lfcShrink() function, specifying “ashr” as the shrinkage estimator. DE genes were defined as genes that had an adjusted *p* value ≤0.05 and log2FC ≥ 0.5. Significance of the overlap between XX vs. XO and XY vs. XO DE genes was calculated using a random sampling method. For XX vs. XO and XY vs. XO, 2,365 and 2,483 genes, respectively, were randomly sampled from the DESeq2 object using the sample() function. The overlap between these two groups of genes was calculated using the intersect() function. This was repeated 100,000 times to generate an expected distribution. The significance of the true overlap was calculated using the pnorm() function, specifying lower.tail = FALSE. The *p* value was corrected using the p.adjust function, specifying “BH” as the correction method. Functional enrichment of DE genes was done as described in San Roman et al. (2024). For the X-to-A ratios, we used the VST counts. As lowly expressed genes had been previously filtered out, all remaining X-linked and autosomal genes were considered for the X-to-A ratio calculation (955 X-linked genes and 17,448 autosomal genes). For each replicate, the median expression value for these X-linked and autosomal genes was calculated using the median() function; the X-to-A ratio was then calculated as X-linked_median/autosomal_median. The results were then visualized as a boxplot. For the XX and XY correlation heatmaps, the samples from this dataset and the Waldhorn dataset were processed together in DESeq2, and the VST counts generated. The XX samples and XY VST counts were separated to make one XX table and one XY table, with each containing the VST counts from both this dataset and the Waldhorn dataset. The top 500 most variable genes per genotype were identified using the rowVars function, thereby ensuring the most variable genes were representative of both datasets. These genes were then used to calculate the correlation scores for the XX and XY samples, using the cor() function and specifying “Pearson” as the correlation method. The heatmap was generated using the correlation scores, using the pheatmap() function from the pheatmap R package (v.1.0.12), specifying cluster_rows = TRUE and cluster_cols = TRUE. For the correlation heatmap containing both datasets and both genotypes together, the XX and XY VST tables were combined rather than separated. The DE gene overlap with the fibroblast data was done using the raw counts fibroblast data from San Roman et al. (San Roman et al., 2024). The raw counts were filtered to keep only XXY, XX, XY, and X genotypes. DESeq2 was used to process the data, specifying “∼batch_libprep + Genotype” as the design formula. Lowly expressed genes were removed, and DE genes were identified and thresholded as described earlier. DE genes from each genotype comparison were then overlapped with the respective comparison from this study. The results were visualized as a Euler plot using the eulerr R package (v.7.0.2).

### Whole-exome sequencing

DNA libraries were prepared using 200 ng of genomic DNA, fragmented to a target size of 150–200 bp on the Covaris E220, as input into an Agilent SureSelect XT library preparation kit, and whole-exome capture was performed using a Human All Exon V5 capture library according to the manufacturer’s guidelines. Libraries were then multiplexed and sequenced using 100 bp paired-end reads on Illumina HiSeq 4000 to a depth of at least 50M paired-end reads per sample.

### SNP identification on X chromosome

For SNP identification, DNA sequence data were trimmed using trim_galore, with the following parameters --paired --fastqc --gzip --retain_unpaired --three_prime_clip_R1 2 --three_prime_clip_R2. Reads were mapped using bwa mem against the human reference hg38 genome using default parameters. Alignment files were converted from sam to bam format, sorted, and indexed using samtools view -b, sort, and index, respectively. PCR doublets were marked using Picard. Variant calling was performed using gatk specifying HaplotypeCaller parameter. The iPSC RNA-seq data were then loaded into IGV (v.2.9.4) to check SNPs in transcribed X-genes. SNPs were classed as informative if they had a read coverage of at least 10.

## Resource availability

### Lead contact

Further information and requests for materials should be directed to and will be fulfilled by the lead contact, James M.A. Turner (james.turner@crick.ac.uk).

### Materials availability

The human autosomally isogenic iPSCs generated in this study are available for research upon completed materials transfer agreement with the Francis Crick Institute. Request for materials should be sent to James M.A. Turner (james.turner@crick.ac.uk).

### Data and code availability

Generated RNA-seq datasets from autosomally isogenic hiPSCS have been deposited at EGA: EGAD50000001361. Generated whole-exome sequencing from skin fibroblasts GM03102 has been deposited at EGA: EGAD50000001362. These datasets are available for research upon completed Data Access Agreement. Request for the code availability should be directed to Wazeer Varsally (wazeer.varsally@crick.ac.uk). Request for data availability should be directed to Human Biology STP at the Crick, human-biology@crick.ac.uk.

## Acknowledgments

This work was supported by AstraZeneca – Crick Collaboration (PRJ_10665) and by 10.13039/100010438Francis Crick Institute, which received its core funding from 10.13039/501100000289Cancer Research UK (CC2052), the 10.13039/501100000265UK Medical Research Council (CC2052), and the 10.13039/100010269Wellcome Trust (CC2052). The authors thank the Francis Crick Institute Human Biology, Advanced Light Microscopy, and Advanced Sequencing facilities for their contributions and expertise. We thank the members of the J.M.A.T. lab for comments and discussion on the manuscript.

## Author contributions

Study conception and design, J.M.A.T. and B.E.P.; investigation and data analysis, R.M.; bioinformatic data analysis, W.V. and J.Z.; manuscript writing and revision, J.M.A.T., R.M., W.V., L.S., T.I., J.E., R.H., and A.P.; supervision, J.M.A.T., R.H., and A.P.

## Declaration of interests

L.S., T.I., J.E., and A.P. are AstraZeneca employees. R.H., J.E., T.I., L.S., and A.P. are AstraZeneca shareholders.

## Supplemental information

Document S1. Figures S1–S3 and Tables S2 and S3

Table S1. RNA-seq analysis of isogenic hiPSCs with different sex chromosome complements (A) Fold change and p-values for all genes between isogenic XX and XY hiPSCs of current study. (B) Gene ontology (GO) analysis of XX versus XY hiPSCs of current study. (C) Fold change and p-values between XXY vs. XX hiPSCs of current study. (D) Fold change and p-values between XXY vs. XY hiPSCs of current study. (E) Fold change and p-values between XY vs. XO hiPSCs of current study. (F) Fold change and p-values between XX vs. XO hiPSCs of current study. (G) Comparison of differentially expressed genes (DEGs) between current and the San Roman study. (H) Overlap between XY vs. XO and XX vs. XO DEGs of current study. (I) GO analysis of shared DEGs between XY vs. XO and XX vs. XO DEGs of current study. (J) List of unique genes between XX vs. XO of current study. (K) List of unique genes between XY vs. XO of current study. (L) GO analysis of unique XX vs. XO DEGs of current study. (M) GO analysis of unique XY vs. XO DEGs of current study.

Document S2. Article plus supplemental information
