# Supplementary material for: A human induced pluripotent stem cell toolbox for studying sex chromosome effects
Source: Stem Cell Reports. 2025 Oct 16;20(11):102678. doi: 10.1016/j.stemcr.2025.102678 (PMC12790746; doi:10.1016/j.stemcr.2025.102678)
Supplement: Document S1. Figures S1–S3 and Tables S2 and S3 [file mmc1.pdf]

Stem Cell Reports, Volume *20*

Supplemental Information

A human induced pluripotent stem cell toolbox for studying sex chromosome effects

Ruta Meleckyte, Wazeer Varsally, Jasmin Zohren, Jerry Eriksson, Tania Incitti, Linda Starnes, Amy Pointon, Ryan Hicks, Benjamin E. Powell, and James M.A. Turner

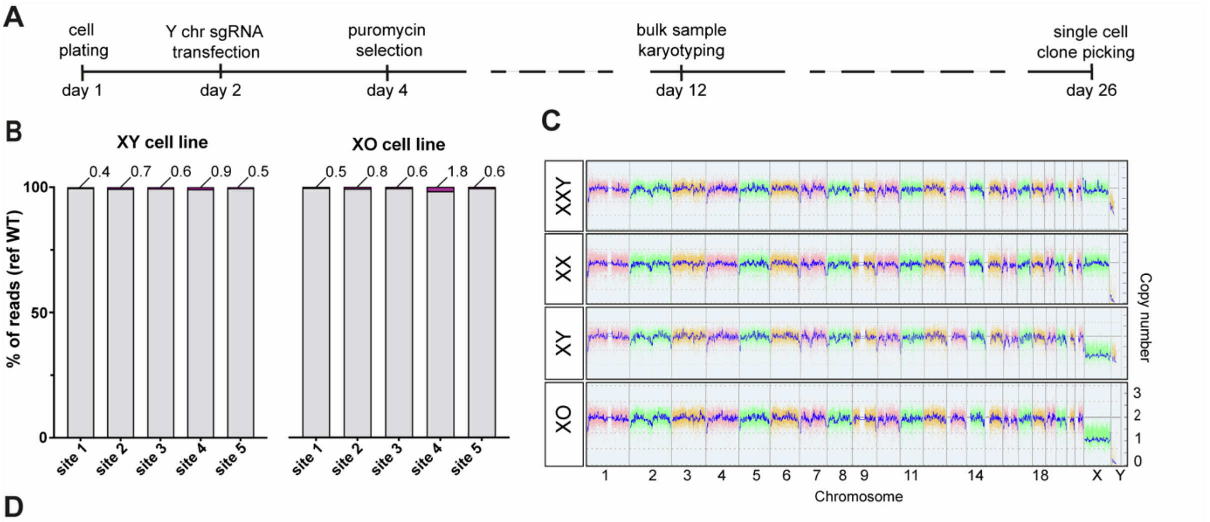

D

X chr gene	X chr coordinate	fib DNA SNP	iPSC SNP inferred from RNA data											
			XXY_1	XXY_2	XXY_3	XX_1	XX_2	XX_3	XY_1	XY_2	XY_3	XO_1	XO_2	XO_3
GSPT2	51744141	C/G	C	C	C	C	C	C	C	C	C	C	C	C
TFE3	49030447	C/G	C	C	C	C	C	C	C	C	C	C	C	C
SYN1	47585586	T/C	T	T	T	T	T	T	T	T	T	T	T	T
ZNF41	47448825	A/C	A	A	A	A	A	A	A	A	A	A	A	A
SLC9A7	46758886	C/G	G	G	G	G	G	G	G	G	G	G	G	G
DYNLT3	37841795	G/A	G	G	G	G	G	G	G	G	G	G	G	G
PDHA1	19361522	G/C	G	G	G	G	G	G	G	G	G	G	G	G
WWC3	10128090	C/T	C	C	C	C	C	C	C	C	C	C	C	C
SHROOM2	9946907	G/C	C	C	C	C	C	C	C	C	C	C	C	C

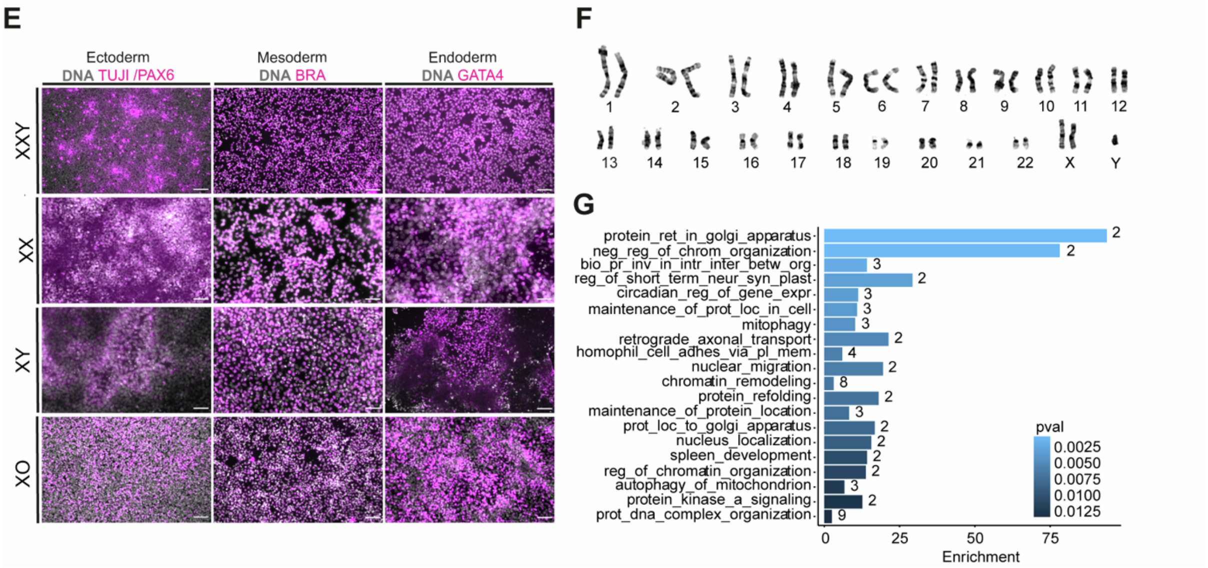

Figure S1. Characterisation of generated autosomally isogenic hiPSC lines and functional enrichment analysis.
A) Schematic view of Y chromosome elimination to generate XO hiPSCs.
B) Analysis of MiSeq reads on XY and XO hiPSCs, focusing on top five off-target sites located in chr4, chr7, chr8, chr11 and chr22. Non-reference reads are marked with magenta.
C) Karyostat analysis of hiPSCs.
D) Active X chromosome SNPs in hiPSCs.
E) Direct differentiation of hiPSCs. Scale bar in all images is 100μm.
F) G-banding on XXY iPSC line.
G) Enrichment analysis of DE genes between XX and XY hiPSCs.

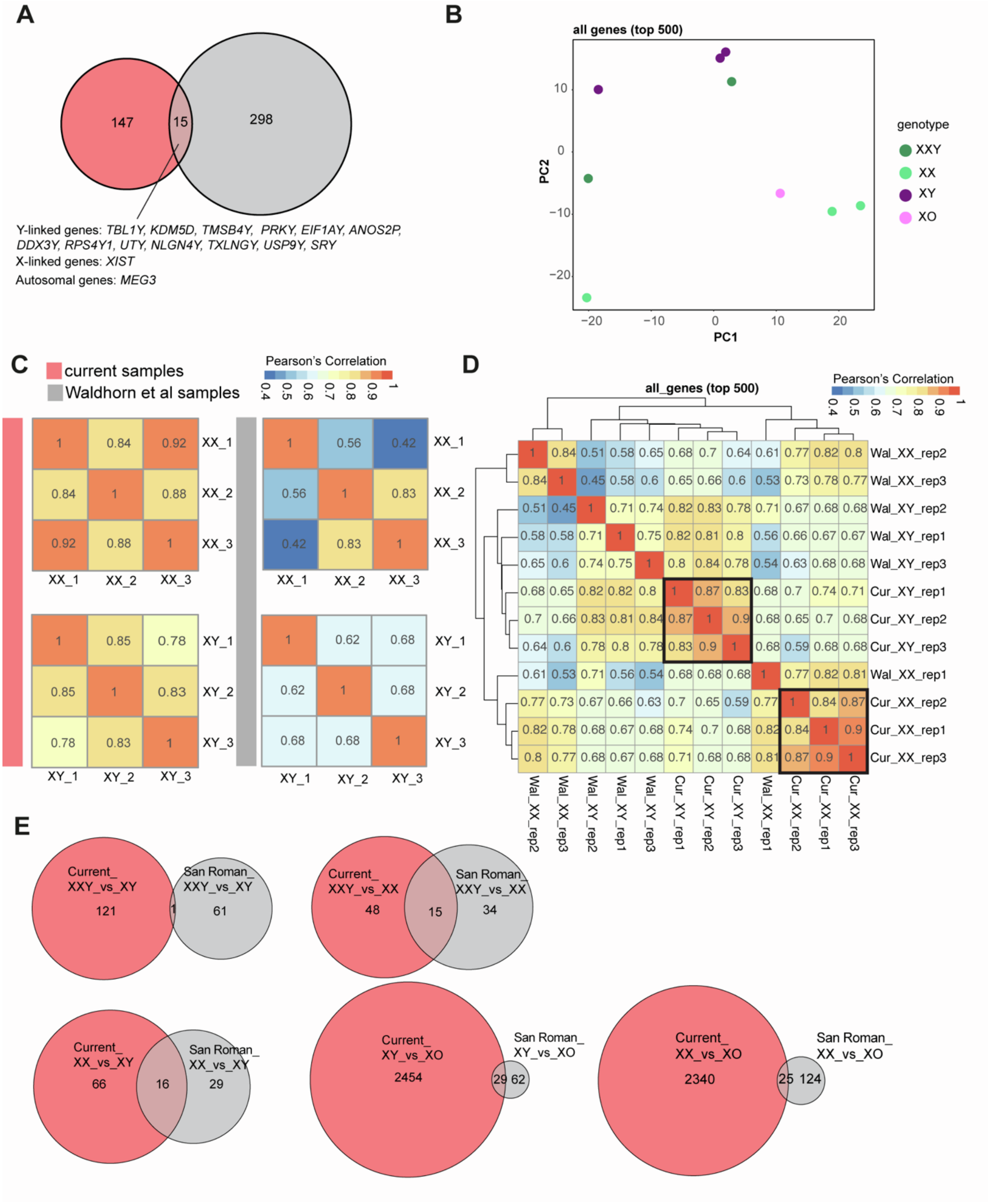

Figure S2. Comparison of the transcriptional landscape of generated hiPSCs with Waldhorn et al generated isogenic hiPSCs and the San Roman et al fibroblasts dataset.

A) Euler diagram of XX vs XY DE genes between this study (pink circle) and the Waldhorn (grey circle) study. 15 common genes between studies are shown.

B) PCA plot using the top 500 most variable genes for the autosomally isogenic iPSCs set from the Waldhorn study.

C) Pearson correlation coefficient (PCC) heatmap between genotypes and replicates. Sample correlations from this study are on the left, Waldhorn study – on the right. Correlations calculated using the top 500 most variable genes in XX and XY genotypes independently.

D) PCC heatmap using the top 500 most variable genes across all autosomally isogenic iPSCs samples. Black boxes highlight the XX and XY replicates generated in this study.

E) Comparison of DE genes between generated hiPSCs (pink circle) and the San Roman et al fibroblasts dataset (grey circle) with various sex chromosome complement.

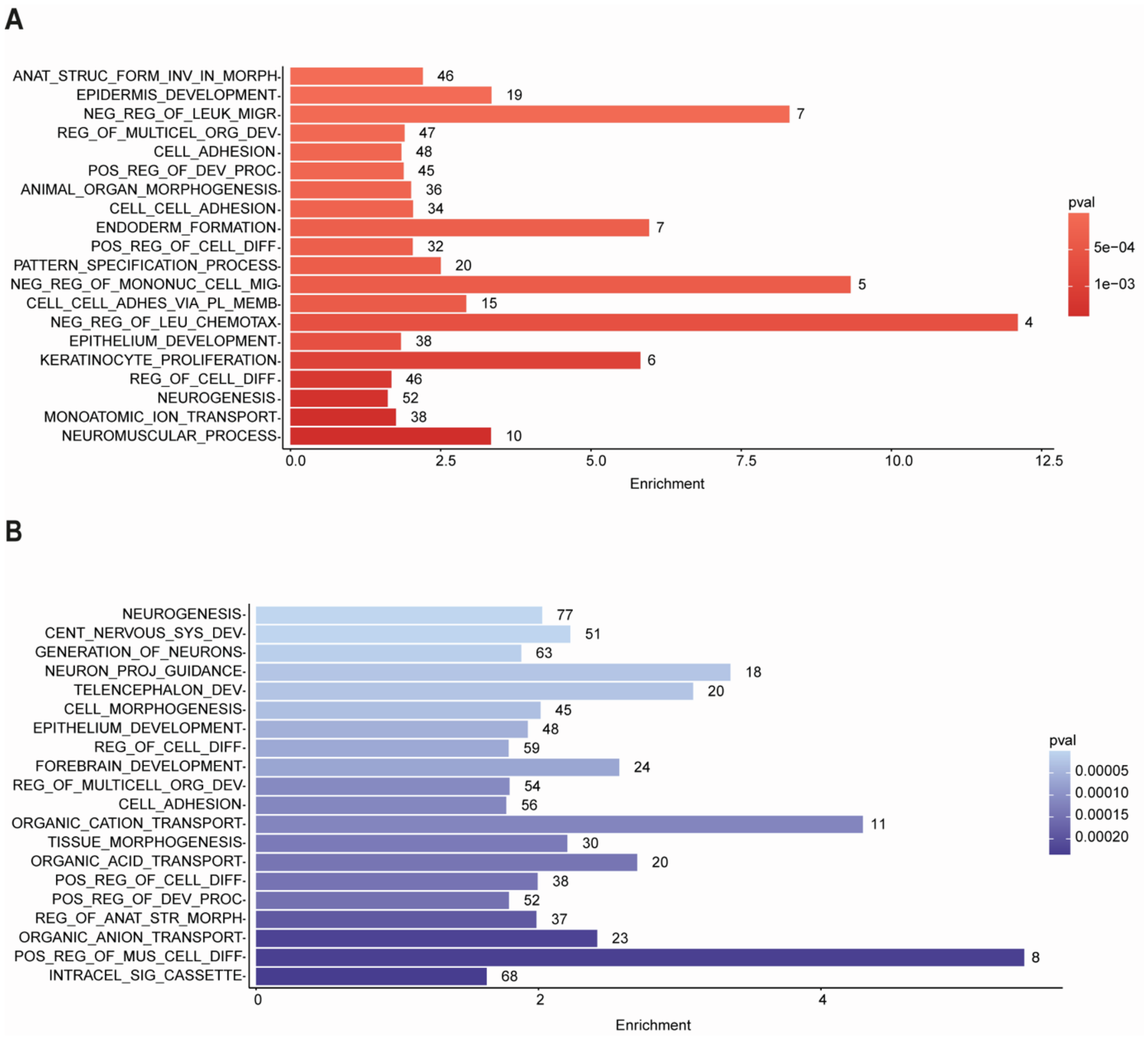

Figure S3. Functional enrichment analysis of DE genes between XO, XX and XY hiPSCs.
A) Enrichment analysis of unique DE genes in the XX versus XO comparison.
B) Enrichment analysis of unique DE genes between in the XY versus XO comparison.

Table S1. RNA-seq analysis of isogenic hiPSCs with different sex chromosome complements. A. Fold change and p-values for all genes between isogenic XX and XY hiPSCs of current study. B. Gene ontology (GO) analysis of XX versus XY hiPSCs of current study. C. Fold change and p-values between XXY vs XX hiPSCs of current study. D. Fold change and p-values between XXY vs XY hiPSCs of current study. E. Fold change and p-values between XY vs XO hiPSCs of current study. F. Fold change and p-values between XX vs XO hiPSCs of current study. G. Comparison of differentially expressed genes (DEGs) between current and the San Roman study. H. Overlap between XY vs XO and XX vs XO DEGs of current study. I. GO analysis of shared DEGs between XY vs XO and XX vs XO DEGs of current study. J. List of unique genes between XX vs XO of current study. K. List of unique genes between XY vs XO of current study. L. GO analysis of unique XX vs XO DEGs of current study. M. GO analysis of unique XY vs XO DEGs of current study.

Table S2. Primers for off-target region sequencing and sgRNA used in this study, related to Figure 1B, and Figure S1B. Capital letters indicate MiSeq adaptor sequence in off-target primers.

Primer	Sequence
hYcen_off_Ch4_F	TCGTCGGCAGCGTCAGATGTGTATAAGAGACAGaatgcgagctgctgaaagat
hYcen_off_Ch4_R	GTCTCGTGGGCTCGGAGATGTGTATAAGAGACAGccccaaccactgtcacttct
hYcen_off_Ch22q_F	TCGTCGGCAGCGTCAGATGTGTATAAGAGACAGaacaccactctcgccatctc
hYcen_off_Ch22q_R	GTCTCGTGGGCTCGGAGATGTGTATAAGAGACAGgggcaacgtagtgagagacc
hYcen_off_Ch11_F	TCGTCGGCAGCGTCAGATGTGTATAAGAGACAGgatgaatgcccacatcagaa
hYcen_off_Ch11_R	GTCTCGTGGGCTCGGAGATGTGTATAAGAGACAGggtttcaggcctatggtgag
hYcen_off_Ch7_F	TCGTCGGCAGCGTCAGATGTGTATAAGAGACAGttttcaccatgggactcaaa
hYcen_off_Ch7_R	GTCTCGTGGGCTCGGAGATGTGTATAAGAGACAGgcatttggggaaatcagaaa
hYcen_off_Ch8_F	TCGTCGGCAGCGTCAGATGTGTATAAGAGACAGgtggaaacggggatacgtta
hYcen_off_Ch8_R	GTCTCGTGGGCTCGGAGATGTGTATAAGAGACAGgagtgcacacaacccaaaga
sgRNA_h_chrY_centr_F	CACCGAAACGATAGTTTCGACTCTG
sgRNA_h_chrY_centr_R	AAACCAGAGTCGAAACTATCGTTTC

Table S3. Antibodies used in this study for hiPSCs undifferentiated and differentiated status confirmations, related to Figure 1F, and Figure S1E.

Antibody	Company	Cat. No.	Dilution
OCT4	Santa Cruz Biotech	sc-5279	1:200
NANOG	R&D systems	AF1997	1:100
BRACHURY	R&D systems	AF2085	1:100
CDX2	abcam	Ab76541	1:100
GATA4	R&D systems	BAF2606	1:100
TUJI	Sigma	T5076	1:100
PAX6	abcam	Ab5790	1:100
ALEXAFLOUR 488 anti-mouse	Thermofisher Scientific	A21200	1:200
ALEXAFLOUR 594 anti-rabbit	Thermofisher Scientific	A21442	1:200
ALEXAFLOUR 647 anti-rabbit	Thermofisher Scientific	A21443	1:200

Supplemental Experimental Procedures

Transfection assay

Human iPSCs were maintained in Advanced E8 or StemFlex medium and plated as single cells a day before transfection. On day 1, cells were transfected with the Cas9-eGFP vector plasmid containing sgRNA targeting Y chromosome, Optimem (Gibco, 31985-062) and FuGeneHD buffer (Promega, E2311). Ratios were used following manufacturer's instructions. Targeted hiPSC clones were selected by adding puromycin (0.5-1 ug/ml) when they've reached 80% confluency on day 2 or day 3 for 24-48 hours. Targeted colonies appeared by day 6-12. Transfection efficiency is dependent on cell line and was recorded between 5 % - 45 %. In this study we use XY_1, XY_2 and XY_3 cell lines, passage numbers between P15 - P18.

Direct differentiation assay

The STEMdiff™ Trilineage Differentiation Kit (StemCell Technologies, 5230) was used to differentiate cells into three germ lineage precursors. The assay was performed as per kit instructions. We evaluated the differentiation potential of all lines by immunostaining for lineage-specific markers on day 5 (Mesoderm and Endoderm) and day 7 (Ectoderm). Cell lines' passage numbers were between P10 - P26 for this experiment.

Primer design

All primer pairs used in this study were designed using the publicly available tool Primer3 (http://bioinfo.ut.ee/primer3/). All PCR amplifications were carried out using Q5 High-Fidelity DNA polymerase (NEB) at recommended Q5 thermocycling conditions. To amplify off target regions for MiSeq analysis, primers were designed using Primer3 and extended to contain MiSeq adaptor sequences (see relevant section). All primer sequences are listed in Table S2.

MiSeq high throughput sequencing

Five off-target sites in chr 4, chr7, chr11 and chr22 were tested by amplifying the regions using MiSeq PCR primers (Table S2.) in a total volume of 25 µl (12.5 µl NEB Q5 High-Fidelity Master Mix, 5 mM each primer). Correct PCR amplification was confirmed by gel electrophoresis. Resultant PCR amplicons were purified using beads AMPure XP (Beckman coulter, A63881) and resuspended in 15 µl nuclease-free water and prepared according to the Illumina MiSeq library prep manufacturer's instructions (Nextera Index Kit V2). Libraries are quantified using Promega Quantifluor reagents, plate reader and Agilent Tapestation. MiSeq libraries are pooled by concentration and sequenced on the Illumina MiSeq-Nano platform with a PE 250bp run configuration on a Nano flowcell. On average samples receive 2,000-5,000 reads each.

Resultant reads were demultiplexed and fastq files were collapsed using FastX Toolkit (v0.0.13) [https://github.com/agordon/fastx_toolkit]. To assess the rate of indel-production by CRISPR-Cas9, the reads were aligned to the human reference genome hg38 with the Burrows-Wheeler Alignment tool (BWA, v0.7.170) (Li and Durbin, 2009) using the mem algorithm with default settings and then analysed using the R package CrispRVariants (v1.14.0) (Lindsay et al., 2016). The proportion of wild type reads in off target sites were calculated by dividing the number of reads matched to reference genome by sum of reference reads and all reads containing single nucleotide variants ± 20bp away from the sgRNA binding site.

Whole Exome Sequencing

DNA libraries were prepared using 200 ng of genomic DNA, fragmented to a target size of 150 to 200 bp on the Covaris E220, as input into an Agilent SureSelect XT library preparation kit, and whole-exome capture was performed using a Human All Exon V5 capture library according to the manufacturer's guidelines. Libraries were then multiplexed and sequenced using 100 bp paired-end reads on Illumina HiSeq 4000 to a depth of at least 50M paired-end reads per sample.

SNP identification on X chromosome

For SNP identification, DNA sequence data were trimmed using trim_galore, with the following parameters --paired --fastqc --gzip --retain_unpaired --three_prime_clip_R1 2 --three_prime_clip_R2 Reads were mapped using bwa mem against the human reference hg38 genome using default parameters. Alignment files were converted from sam to bam format, sorted and indexed using samtools view -b, sort and index respectively. PCR doublets were marked using Picard. Variant calling was performed using gatk specifying HaplotypeCaller parameter. The iPSC RNA-seq data was then loaded into IGV (v2.9.4) to check SNPs in transcribed X-genes. SNPs were classed as informative if they had a read coverage of at least 10.

Supplemental Reference

Li, H., & Durbin, R. (2009). Fast and accurate short read alignment with Burrows-Wheeler transform. *Bioinformatics*, *25*(14), 1754–1760. https://doi.org/10.1093/bioinformatics/btp324.

Lindsay, H., Burger, A., Biyong, B., Felker, A., Hess, C., Zaugg, J., Chiavacci, E., Anders, C., Jinek, M., Mosimann, C., & Robinson, M. D. (2016). CrispRVariants charts the mutation spectrum of genome engineering experiments. In *Nature Biotechnology* (Vol. 34, Issue 7, pp. 701–702). Nature Publishing Group. https://doi.org/10.1038/nbt.3628.

San Roman, A. K., Skaletsky, H., Godfrey, A. K., Bokil, N. V., Teitz, L., Singh, I., Blanton, L. V., Bellott, D. W., Pyntikova, T., Lange, J., Koutseva, N., Hughes, J. F., Brown, L., Phou, S., Buscetta, A., Kruszka, P., Banks, N., Dutra, A., Pak, E., … Page, D. C. (2024). The human Y and inactive X chromosomes similarly modulate autosomal gene expression. *Cell Genomics*, *4*(1). https://doi.org/10.1016/j.xgen.2023.100462.

Waldhorn, I., Turetsky, T., Steiner, D., Gil, Y., Benyamini, H., Gropp, M., & Reubinoff, B. E. (2022). Modeling sex differences in humans using isogenic induced pluripotent stem cells. *Stem Cell Reports*, *17*(12), 2732–2744. https://doi.org/10.1016/j.stemcr.2022.10.017.
